# Hybrid-Total Ankle Arthroplasty (H-TAA) for Failed Talar Component in Mobile-Bearing Total Ankle Arthroplasty

**DOI:** 10.3390/jcm12051764

**Published:** 2023-02-22

**Authors:** Simone Santini, Waheeb Alharbi, Kar Hao Teoh, Mario Herrera-Perez, Victor Valderrabano

**Affiliations:** 1 Department of Orthopaedic and Trauma Surgery, University Campus Bio-Medico of Rome, 00128 Rome, Italy; 2 King Fahad Armed Forces Hospital, Al Kurnaysh Rd, Al Andalus, Jeddah 23311, Saudi Arabia; 3 Princess Alexandra Hospital NHS Trust, Harlow CM20 1QX, UK; 4 Head Foot and Ankle Unit, Orthopaedic Department, Universidad de La Laguna, 38200 San Cristóbal de La Laguna, Spain; 5 Swiss Ortho Center, Swiss Medical Network, Schmerzklinik Basel, Hirschgässlein 15, 4010 Basel, Switzerland

**Keywords:** ankle, foot, Total Ankle Arthroplasty, Total Ankle Replacement, revision, mobile bearing, Flatcut Talus

## Abstract

Introduction: Revision Total Ankle Arthroplasty (TAA) surgery due to TAA aseptic loosening is increasing. It is possible to exchange the talar component and inlay to another system for isolated talar component loosening in a primary mobile-bearing TAA: Hybrid-Total Ankle Arthroplasty (H-TAA). The purpose of this study was to analyze the results of the revision surgery of an isolated aseptic talar component loosening in a mobile-bearing three-component TAA with a H-TAA solution. Methods: In this prospective case study, nine patients (six women, three men; mean age 59.8 years; range 41–80 years) with symptomatic isolated aseptic loosening of the talar component of a mobile-bearing TAA were treated with an isolated talar component and inlay substitution. In all nine cases, a hybrid TAA revision surgery was performed by implanting a VANTAGE TAA talar and insert component (Flatcut talar component: six cases, standard talar component: three cases). The patients were reviewed with the pain score (VAS Pain Score 0–10), Dorsiflexion/Plantarflexion (DF/PF) Range of Motion (ROM; degrees), the American Orthopaedic Foot and Ankle Society (AOFAS) Ankle/Hindfoot Score (0–100 points), Sports Frequency Score (Level 0–4), and subjective Patients’ Satisfaction Score (0–10 points). Results: The average Pain score improved significantly from preoperative 6.7 points to postoperative 1.1 points (*p* < 0.001). Average Dorsiflexion/Plantarflexion ROM values increased significantly post-surgery: 21.7° preoperative to 45.6° postoperative (*p* < 0.001). The postoperative AOFAS scores were significantly greater than the preoperative values: 47.7 points preoperative, 92.3 points postoperative (*p* < 0.001). The sports activity improved from preoperative to postoperative where, preoperative, none of the patients were able to perform sports. Postoperative, eight patients were able to be sports-active again. The overall average postoperative level of sports activity was 1.4. The postoperative average patient’s satisfaction was 9.3 points. Conclusions: In painful talar component aseptic loosening of a three-component mobile-bearing TAA, H-TAA is a good surgical solution for reducing pain, restoring ankle function, and improving patients’ life quality.

## 1. Introduction

Ankle Osteoarthritis (OA) is a condition characterized by pain, swelling, and inflammation associated with chronic, progressive, and irreversible damage to articular cartilage [1]. The most common etiology is post-traumatic (78%), followed by secondary causes such as rheumatoid arthritis, hemophilia, hemochromatosis (13%), and primary degeneration of the joint, which accounts for only 9% of cases [2,3,4].

Ankle OA leads to severe functional limitations in everyday life and sports activity. The level of physical and psychological disability associated with ankle OA is at least as severe as that of hip OA [5].

Surgical treatments of ankle OA can be divided into joint preserving and joint sacrificing surgical procedures [1]. Patients who have incomplete asymmetric ankle OA, significant daily pain, mild to moderate functional limitations, good bone quality, and asymmetric alignment of the lower limbs—associated or unassociated with joint instability—may benefit from joint preserving surgery, usually a supramalleolar osteotomy of the tibia/fibula (SMOT). It is typically recommended for young patients and those without systemic comorbidities. Its objective is to restore the joint’s stability, alignment, and biomechanics and to delay more intrusive operations for five to ten years by slowing the progression of joint degeneration at the most damaged compartment [4].

The two main therapeutic options for end-stage ankle OA are Total Ankle Arthroplasty (TAA) and ankle arthrodesis. Ankle arthrodesis, once considered the gold standard, was previously recommended in most patients because of its predictable outcomes and decreased complication rates [6]. This therapeutic decision-making process was changed by the increasing survivorship rate and positive results of TAA, which at the very least produced improved biomechanical and functional results [1,7]. In a 2021 meta-analysis, a better health-related quality of life was found in patients who underwent TAA than ankle arthrodesis [8]. The TAA group also improved AOFAS score/function and had better ROM. With high satisfaction ratings, TAA can enhance function while preserving ankle mobility and reducing pain [8]. After receiving treatment from TAA, the total sports participation rate considerably rose, along with the patient’s quality of life [9].

A recent meta-analysis showed that at a 10-year follow-up there is a TAA survival rate of 70 to 90% and an average 10-year TAA revision rate of 22% [10]. Every implant has shown different survivorship: Hintegra has a probability of survival of components of 93.5% at the mean follow-up of 11.3 years (range, 120.0 to 204.0 months) [11]; STAR showed a survivorship of 70.7% after 10 years, 45.6% after 14 years [12]; Agility has shown a 76% of survivorship after 9 years [13]; Salto implant has shown a survivorship of 98% after 5 years [14] and 85% after 10 years [15].

Aseptic loosening is the most frequent complication following TAA, accounting for 55% of failures along with subsidence of the talar component into the talar body [16,17,18,19]. This peri-implant osteolytic process is characterized by particle wear debris produced by mechanical interaction between implant components [17].

Four surgical options are feasible for managing a TAA failure.

Two-staged revision is where the loose TAA is removed during the first stage while simultaneously restoring the bone stock. This is followed by verification of a successful restoration of bone stock via CT-scan and while proceeding to the second stage of implanting a new TAA [20].

Primary Hybrid TAA (H-TAA) is where a one-stage revision is carried out in selected cases with isolated talar or tibial component loosening. Only the loosened component is changed, and, simultaneously, the bone stock is restored if needed. This is possible only for the loosened talar component if a mobile-bearing TAA system has been used.

Explantation of the failed primary implant and implantation of a TAA Revision System or patient-designed revision TAA is another option. Currently, the only systems available on the market are the INBONE prosthesis and the Salto Talaris XT Revision system. The INBONE device utilizes a lengthy modular tibial stem with intramedullary referencing and fixation [21], while the Salto Talaris XT Revision consists of a tibial component with a long keel and three types of talar component (i.e., flat cut, short stem; flat cut, long stem; and sloped cut, long stem [22]. While a mid-term study (mean follow-up of 40 months) has shown an INBONE II implant survival rate of 97% [23], no long-term outcomes studies have been conducted so far.

In cases of severe prosthesis failure, loosening, subsidence, or a substantial structural bone post-TAA-arthrodesis (ankle or tibiotalocalcaneal arthrodesis) can be performed. The outcome of the structural bone transplant arthrodesis is unpredictable, and the success rate is, at best, low [21]. The goal of such a surgery is bipedal walking and the avoidance of lower leg amputation.

Complete extraction of implants from both joint surfaces, especially in cases where the problem resides on only one component of the prosthesis, would result in unnecessary bone loss that increases the difficulty of the surgery and decreases the likelihood of success. When faced with similar situations in hip and knee arthroplasty, the solution is to use cross-compatible components, possibly from different brands, to protect a well-fixed implant and to form a hybrid hip/knee joint system through partial component revision [24,25].

This paper aims to evaluate clinical and radiological outcomes of a TAA revision surgery by a hybrid total ankle arthroplasty (H-TAA) in a failed talar component of a mobile bearing-TAA at a one-year follow-up.

## 2. Materials and Methods

In this prospective case series with a minimal 1-year follow up, 9 patients (6 women, 3 men; mean age 59.8 years; range 41–80 years) with symptomatic aseptic loosening of the talar component of a mobile-bearing TAA were treated with isolated talar component and polyethylene insert substitution.

The inclusion criteria of the study were painful, isolated, aseptic loosening of the talar component of a mobile TAA with a stable tibial component (at least 70–80% normal tibial bone–implant interface). The exclusion criteria were as follows: loosening of the tibial component, significant non-reconstructable bone defect of the loosened talus, and septic loosening. Table 1 illustrates the demographics of the cohort and the failed primary TAA systems. The underlying ankle OA etiology was posttraumatic ankle OA in 7 cases (78%) and secondary ankle OA in 2 cases (22%) (Table 1). In all the cases, the failed talar component was part of a Hintegra Mobile TAA with survivorship of the revised implant of 7.8 years (range 3–18 years; Table 1).

In all the nine cases, the hybrid TAA was performed through the implantation of a VANTAGE TAA Talar Component (VANTAGE Total Ankle Arthroplasty System, Exactech, Gainesville, FL, USA) with Flatcut Talar Component (six cases) and Standard Talar Component (three cases) (Table 2). All surgeries were performed by the senior author (V.V.).

**Figure 1:**
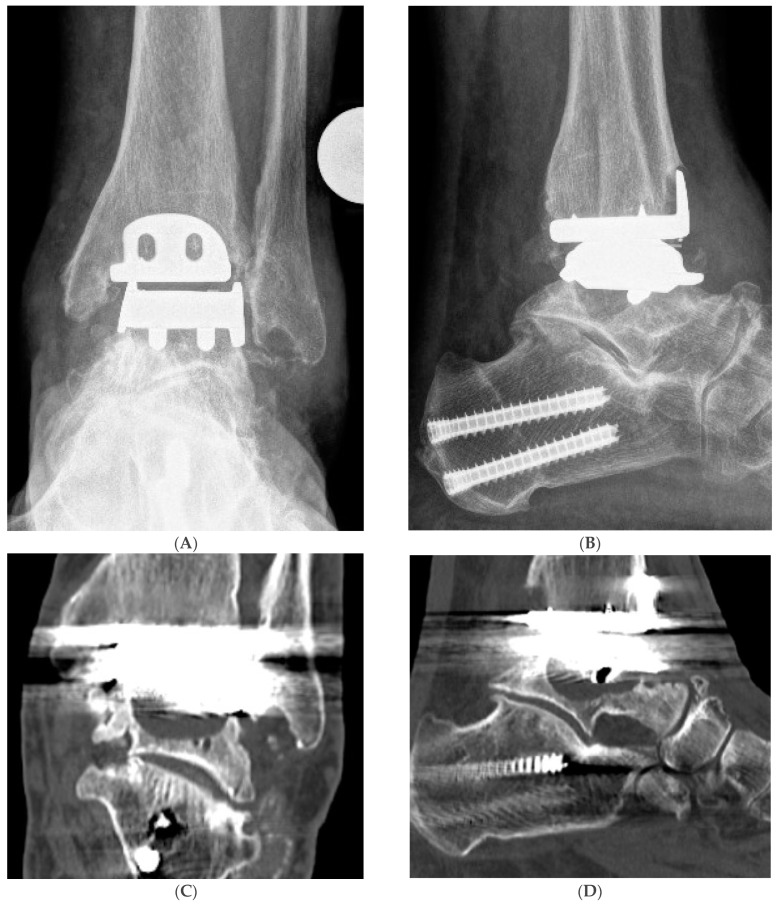

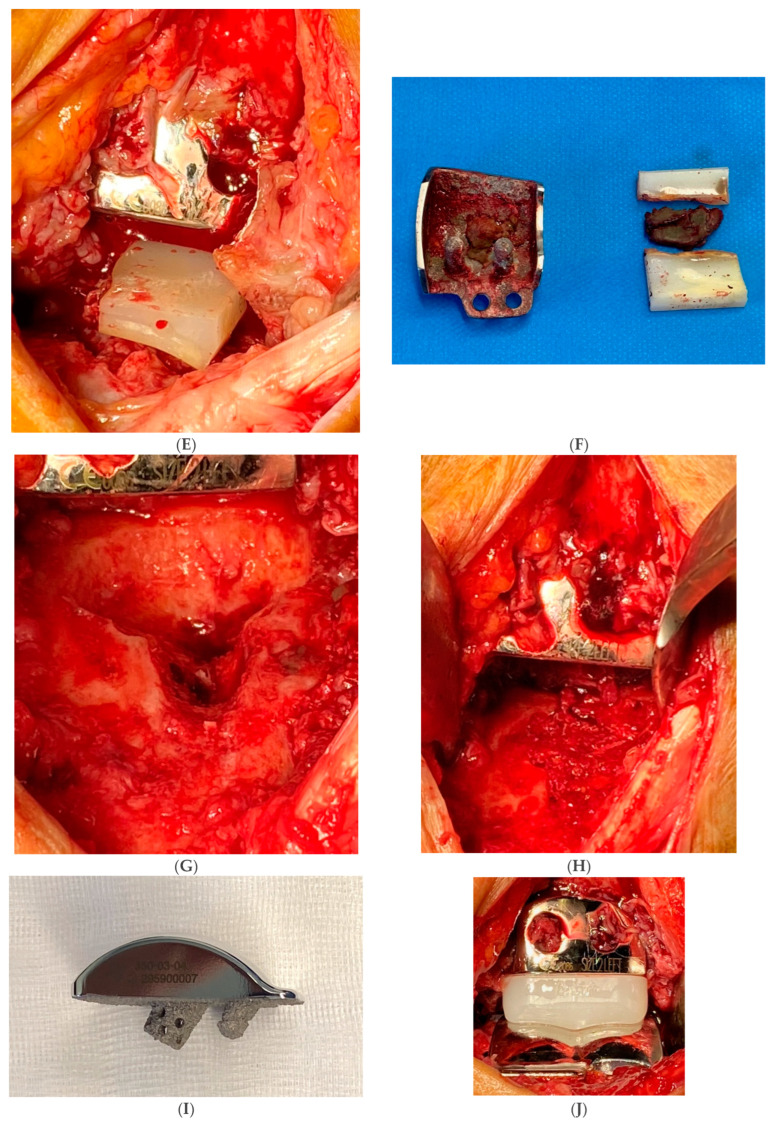

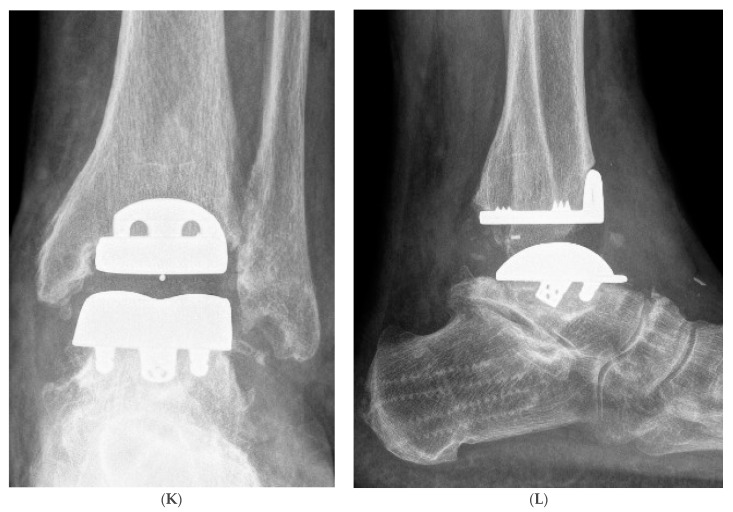
Hybrid Total Ankle Arthroplasty (H-TAA). This figure represents Case Nr. 7 of the cohort (see Table 1, Table 2 and Table 3). (**A**–**D**): Preoperative X-rays and CT-scans depicting the cyst under the talar component of the Hintegra TAA. (**E**,**F**): Intraoperative pictures showing the inlay breakage with poor bone osteointegration in the backside of the talar component of the Hintegra TAA. (**G**): Talar cyst with stable tibial Hintegra component. (**H**): Performance of a flatcut of the talus and bone grafting of the talar cyst with iliac crest autologous spongiosa. (**I**): Flatcut Talar Component of the VANTAGE TAA, which is implanted on the reconstructed talus. (**J**): H-TAA: Tibial component Hintegra TAA with new Flatcut Talus and Inlay of VANTAGE TAA. (**K**,**L**): Postoperative X-rays with H-TAA, good talar bone reconstruction, and good integration of the Flatcut Talus of VANTAGE TAA.

All the patients were scored clinically and radiologically before the revision H-TAA surgery. Clinically, pain (VAS Pain Score 0–10), Dorsiflexion/Plantarflexion (DF/PF) Range of Motion (ROM; degrees), the American Orthopaedic Foot and Ankle Society (AOFAS) Ankle/Hindfoot Score (0–100 points), and Sports Frequency Score [9] (level 0–4: level 0: No sports activity; level 1: Moderate level of sports activity in leisure time, <1 h/week; level 2: Normal level of sports activity in leisure time, 1–5 h/week; level 3: High level of sports activity in leisure time, >5 h/week; level 4: Professional level of sports activity, elite athlete) were documented. There were no preoperative signs of infection in all the cases: redness, pus, or sinus. Dorsoplantar, latero–lateral and Saltzman view X-rays were performed to study grossly the TAA, detect possible cystic formations, foot and ankle associated deformities, and the overall ankle alignment (Figure 1). A Computer Tomography CT or SPECT-CT analysis was required to assess the amount of bone loss and to rule out any tibial component loosening.

At a minimum 1-year postoperative follow-up after H-TAA implantation, the following data were documented: pain score, DF/PF-ROM, AOFAS Ankle/Hindfoot score, Patient’s Satisfaction (points 0–10), Sports Frequency Score.

Weight-bearing radiographs were taken at the 1-year follow-up to document the talar component osteointegration and to rule out any possible sign of tibial component loosening.

To determine whether functional outcomes and scores had improved one year after surgery, data from preoperative and postoperative periods were compared using a 2-sample *t*-test. The cutoff for statistical significance was *p* < 0.05. Radiographic and statistical analysis were performed by the author S.S.

### Surgical Procedure

All the procedures (revision TAA surgery and additional surgeries) were performed in one stage. To exclude a possible infection/septic loosening, a Synovasure alpha defensin test (sensitivity of 69% and a specificity of 94%) with the joint fluid was performed [26]. If the Synovasure test were positive, a cultural biopsy sample would be taken and the complete TAA including the tibial component would be removed. In all cases of the cohort, the Synovasure test was negative. Therefore, the TAA revision surgery with H-TAA was possible in all cases.

A standard anterior approach was made on the previous surgical scar. To preserve the anterior neurovascular bundle, which typically lies behind the tendon of the extensor hallucis longus muscle (EHL) or between the tendons of the extensor hallucis longus and extensor digitorum longus muscles (EDL), depth preparation is carried out lateral of the tibialis anterior tendon.

In most cases, periarticular ossifications (PAO) removal was needed to free up the joint. This PAO autologous bone was collected for possible grafting of talar cysts. Polyethylene (PE) wear-induced synovitis and osteolysis were often detected. The mobile PE insert was removed and then the talar component was explanted. The stability of the tibial component was verified intraoperatively. A meticulous curettage of the aseptic loosening cysts of the talus was performed. At this point, an on-table decision was made to determine whether the bone loss was significant enough to use a VANTAGE Flatcut Talus or acceptable to use a VANTAGE Standard Talar component. The procedure is still possible even if there is substantial central talar bone loss, as the talar cortical ream is available [21]. The talar bone is prepared with the jigs of the VANTAGE TAA System with continuous protection of the implanted old tibial component (plastic protection sleeve) and under fluoroscopy control. A trial was performed with trial components before the implantation of the definitive components. Autologous PAO ankle bone or iliac crest spongiotic bone graft were the main choices to fill the talar defects. Following bone grafting, the VANTAGE definitive talar implant (Flatcut or standard) and the Talus-Component matching VANTAGE Polyethylene Mobile Insert were implanted (Figure 1). After finalizing the TAA ankle revision surgery, further possible additional surgeries were carried out if needed, e.g., Gastrocnemius–Soleus Release. To conclude the surgery, an intra-articular drain was inserted in the ankle joint (for drainage 24 h) and the approach was closed. Table 2 summarizes the surgery of all patients.

## 3. Results

Table 3 summarizes the results of the preoperative and postoperative outcomes following the revision H-TAA. The Pain score on average improved significantly from preoperative 6.7 points to postoperative 1.1 points (*p* < 0.001). Dorsiflexion/Plantarflexion ROM values increased significantly post-surgery on average: 21.7° preoperative to 45.6° postoperative (*p* < 0.001). The postoperative AOFAS scores were significantly greater than the preoperative values: 47.7 points preoperative, 92.3 points postoperative (*p* < 0.001). The sports activity improved from preoperative to postoperative where none of the patients could perform sports preoperatively. Postoperatively, eight patients were able to perform some sports activity. The postoperative overall average level of sports activity was 1.4. The postoperative average patients’ satisfaction was 9.3 points.

The postoperative H-TAA X-rays at follow-up showed a good talar bone reconstruction and good osteointegration of the new VANTAGE talar component in all cases (no radiolucency, no loosening). The existing tibial components were unchanged and showed no loosening. No complications or reoperations for any of the included patients were noticed.

## 4. Discussion

In the present study, all revision Hybrid Total Ankle Arthroplasty (H-TAA) patients showed better results in the Vas pain Score, Dorsiflexion/Plantarflexion Range of Motion, AOFAS Ankle/Hindfoot Score, and sports activity at the 12-month follow-up. The H-TAA solution also noticed high personal satisfaction (Table 3).

Aseptic prosthetic implant failure is frequently due to abnormal tissue reactions (bone and soft tissue) induced by implant-derived material components of wear debris [27,28]. According to recent studies, the presence of periprosthetic osteolysis in TAA is found to be between 35% and 37% at 3–4 years after implantation [29,30,31]. Implantation of a prosthesis generates localized necrosis of surrounding tissues. As a result of this process, reparative granulomatous tissue develops, forming a natural envelope to house the implant components. This envelope, also called the pseudomembrane, contains significant levels of giant-cell and foreign-body macrophage. By macrophage phagocytosis, polyethylene particles are consumed. Therefore, cytokines are generated, which modify osteoblast and osteoclast activity and promote osteolysis [32].

Polyethylene wear, rate of wear, joint size, micromotion, high fluid pressure, and genetics are the main factors responsible for periprosthetic osteolysis [32].

While the danger of periprosthetic osteolysis brought on by the wear of particles is decreased by the use of highly cross-linked polyethylene [33], it may be facilitated through micromotion at the bone-implant contact [34]. To minimize micromotion and, hence, prevent any early loosening, the initial stability and alignment obtained after surgery are essential [32]. With better results and techniques for TAA, there is predicted TAA implantation growth, with a consequent increase of failures and revisions [35]. The ankle arthrodesis, which was long seen as the gold-standard salvage treatment for failed TAA, leaves patients unsatisfied and with functional limitations [36,37,38]. Compared to arthrodesis, revision arthroplasty shows better short-term survival statistics with an increase in PROMS [39].

In a 2019 study, Lachman et al. identified talar component subsidence/loosening as responsible for 36% of metal component failures in total ankle arthroplasty [40]. There are three surgical choices for replacing a failed talar component: (a) replacing it with a standard component of the same design, (b) replacing it with a standard component of a different design, or (c) replacing it with a patient-specific talar component (e.g., whole-talus prosthesis) [21]. Only some of the systems are still in commerce, and not all allow inserting a talar component to manage bone loss and achieve the optimal weight distribution on the remaining talus without changing the tibial component. To our knowledge, only a two-case report by Kharwadkar and Harris explored the possibility of a Hybrid revision if a mobile-bearing TAA system has been used [41]. In TAA with aseptic loosening of the talar component and a stable tibial component (with no or little bone loosening; 70–80% must be solid at the tibial bone–implant interface), isolated talar component revision is possible, saving bone stock for further surgeries. The VANTAGE Talus Flatcut solution allowed the restoration of a plain and solid surface. According to Hintermann and colleagues, components with a flat undersurface perform better in revision surgery [42].

Notwithstanding, when possible, a standard talar component has been used to preserve as much talar bone stock as possible. In mobile-bearing, three-component TAA systems, it is possible to keep the old tibial component and implant a talar component and an inlay of another system: Hybrid Total Ankle Arthroplasty (H-TAA). This is possible as the tibia component and the PE insert are both flat in all mobile-bearing three-component systems. This unique advantage of cross-brand hybridization is only possible in mobile-bearing three-component TAA systems. Suppose that a fixed-bearing, two-component TAA has an isolated talar component failure and the stable tibial component and the TAA system are no longer available in the market or the system has a lower quality. In that case, a H-TAA revision surgery is not possible, and the whole TAA, including the good tibial component, must be explanted.

The present study’s limits are the small cohort of patients and the relatively short-term follow-up. However, this paper is the first paper with the biggest cohort of H-TAA. We believe a one-year follow-up is strong enough to show bone integration to a TAA [7]. However, further long-term results with bigger patient cohorts are needed to confirm the results of this H-TAA revision technique.

In conclusion, Hybrid-Total Ankle Arthroplasty (H-TAA) is an excellent and feasible solution for mobile-bearing TAA revision with isolated talar aseptic loosening. H-TAA spares the patient the use of more invasive TAA revision systems or ankle arthrodesis. H-TAA improves pain, range of motion, function, and sports activity, with high patient satisfaction.

## Figures and Tables

**Table 1:** Patients with Partial Failure of the Mobile-Bearing Total Ankle Arthroplasty (TAA), Failed Talar Component.

Patient	Gender (Female, Male)	Age (Years)	Ankle OA Etiology (Before Primary TAA Implantation; Posttraumatic, Secondary, Primary)	Failed Mobile TAA System	Survivorship of Failed TAA (Years)
Nr. 1	Female	63	Posttraumatic	Hintegra–Talus component	3
Nr. 2	Female	74	Secondary	Hintegra–Talus component	18
Nr. 3	Female	41	Posttraumatic	Hintegra–Talus component	3
Nr. 4	Male	80	Secondary	Hintegra–Talus component	4
Nr. 5	Male	62	Posttraumatic	Hintegra–Talus component	4
Nr. 6	Female	65	Posttraumatic	Hintegra–Talus component	11
Nr. 7	Female	62	Posttraumatic	Hintegra–Talus component	4
Nr. 8	Male	50	Posttraumatic	Hintegra–Talus component	13
Nr. 9	Female	41	Posttraumatic	Hintegra–Talus component	10
Average	Female: n = 6 Male: n = 3	59.8 years (range, 41–80 years)	Posttraumatic: n = 7 Secondary: n = 2	Hintegra–Talus component: n = 9	7.8 years (range, 3–18 years)

TAA: Total Ankle Arthroplasty, OA: Osteoarthritis.

**Table 2:** Revision Total Ankle Arthroplasty (TAA) Surgery with Hybrid-TAA (H-TAA).

Patient	New Implanted Vantage TAA System Components (Flatcut Talus and Inlay; Standard Talus and Inlay)	Talus Bone Grafting (Yes from PAO/Iliac Crest; None)	Additional Surgeries
Nr. 1	Standard Talus and Inlay	Iliac crest	PAO Resection Gastrocnemius-Soleus Release (Strayer)
Nr. 2	Standard Talus and Inlay	PAO	PAO Resection
Nr. 3	Standard Talus and Inlay	PAO	PAO Resection
Nr. 4	Flatcut Talus and Inlay	PAO	PAO Resection, release of deltoid ligament
Nr. 5	Flatcut Talus and Inlay	Iliac crest	PAO Resection, Dwyer calcaneus osteotomy, lateral ankle ligament reconstruction, reversed Cotton osteotomy
Nr. 6	Flatcut Talus and Inlay	Iliac crest	PAO Resection, Gastrocnemius-Soleus Release (Strayer)
Nr. 7	Flatcut Talus and Inlay	Iliac crest	Hardware removal
Nr. 8	Flatcut Talus and Inlay	Iliac crest	PAO Resection, cyst bone filing medial malleolus and dorsal tibial component
Nr. 9	Flatcut Talus and Inlay	PAO	PAO Resection
Overall	Flatcut Talus and Inlay: n = 6 Standard Talus and Inlay: n = 3	Iliac crest: n = 5 PAO: n = 4	

TAA: Total Ankle Arthroplasty; PAO periarticular ossification.

**Table 3:** Results of Hybrid Total Ankle Arthroplasty (H-TAA).

	Preoperative				Follow-Up					
Patient	P-Pain (Points 0–10)	P-DF-PF-ROM (Degrees; Sum of DF and PF)	P-AOFAS-Score (Points 0–100)	FU-Sports Frequency Score (Level 0–4)	FU-Time After H-TAA (Months)	FU-Pain (Points 0–10)	FU-DF-PF-ROM (Degrees; Sum of DF and PF)	FU-AOFAS-Score (Points 0–100)	FU-Sports Frequency Score (Level 0–4)	FU-Patient’s Satisfaction (Points 0–10)
Nr. 1	7	20°	40	0	24	0	40°	92	2	10
Nr. 2	5	30°	56	0	24	0	40°	94	1	10
Nr. 3	6	25°	40	0	24	4	50°	74	2	8
Nr. 4	7	20°	58	0	12	2	40°	90	0	9
Nr. 5	8	40°	34	0	12	0	60°	100	2	10
Nr. 6	7	10°	43	0	12	1	40°	92	1	8
Nr. 7	8	20°	30	0	12	0	65°	100	2	10
Nr. 8	5	20°	77	0	12	3	40°	93	2	9
Nr. 9	7	10°	51	0	12	0	35°	96	1	10
Average	6.7	21.7°	47.7	0	16	1.1	45.6°	92.3	1.4	9.3
Range	5–8	10–40°	30–77	0	12–24	0–4	35–65°	74–100	0–2	8–10
*p*-Value						*p* < 0.001	*p* < 0.001	*p* < 0.001	*p* < 0.001	*p* < 0.001

P: Preoperative; DF: Dorsiflexion of ankle; PF: Plantarflexion of ankle; AOFAS: American Orthopaedic Foot and Ankle Society (AOFAS) Ankle/Hindfoot Score; FU: Follow-up; *p*-value: significance difference of *p*-value between postoperative and preoperative variable.

## Data Availability

The data presented in this study are available on request from the corresponding author. The data are not publicly available for ethical and privacy reasons.
